# Supplementary material for: Identification of Inhibitors to Trypanosoma cruzi Sirtuins Based on Compounds Developed to Human Enzymes
Source: Int J Mol Sci. 2020 May 22;21(10):3659. doi: 10.3390/ijms21103659 (PMC7279216; doi:10.3390/ijms21103659)
Supplement: Supplementary file 1 [file ijms-21-03659-s001.pdf]

Supplementary Tables

Table S1. Comparative analysis of amino acid sequence of *T. cruzi* vs human sirtuins (% identity).

	SIRT1	SIRT2	SIRT3	SIRT4	SIRT5	SIRT6	SIRT7
TcSir2rp1	14	29	24	18	20	19	17
TcSir2rp3	7	14	17	23	28	16	13

Table S2. Comparison of Smina and Plants docking software

**Docking Program**	**Scoring Function**	**DA%**	
		ECRD	RCRD
Smina	AD4_SCORING	41.3	45.7
	AD4_SCORING*	50.0	50.0
	VINARDO	69.6	56.5
	VINARDO*	69.6	52.2
	VINA	73.9	56.5
	VINA*	73.9	67.4
Plants	CHEMPLP	47.8	58.7
	PLP95	34.8	43.5
	PLP	54.3	56.5
* after using the minimization feature			

Table S3. Molecular docking numerical results for the active MC inhibitors.

Inhibitor	Protein	Cofactor	PredictedAffinity (kcal/mol)
**15**	TcSir2RP1	NAD	-8.9
		ADP-ribose	-8.0
		Apoprotein	-7.7
Z-**17**	TcSir2RP1	NAD	-11.6
		ADP-ribose	-10.2
		Apoprotein	-10.3
E-**17**	TcSir2RP1	NAD	-11.5

		ADP-ribose	-11.0
		Apoprotein	-10.9
Z-*R*-**8**	TcSir2RP3	NAD	-10.2
		ADP-ribose	-8.1
		Apoprotein	-11.0
Z-*S*-**8**	TcSir2RP3	NAD	-10.3
		ADP-ribose	-7.7
		Apoprotein	-12.2
E-*R*-**8**	TcSir2RP3	NAD	-10.1
		ADP-ribose	-9.4
		Apoprotein	-11.3
E-*S*-**8**	TcSir2RP3	NAD	-10.1
		ADP-ribose	-9.1
		apoprotein	-11.1
**13**	TcSir2RP3	NAD	-10.5
		ADP-ribose	-8.3
		Apoprotein	-12.2
**12**	TcSir2RP3	NAD	-12.4
		ADP-ribose	-9.1
		Apoprotein	-13.0
Z-**30**	TcSir2RP3	NAD	-8.3
		ADP-ribose	-6.8
		Apoprotein	-9.4
E-**30**	TcSir2RP3	NAD	-8.1
		ADP-ribose	-6.8
		Apoprotein	-9.5
Z-**31**	TcSir2RP3	NAD	-8.5
		ADP-ribose	-6.8
		Apoprotein	-9.2
E-**31**	TcSir2RP3	NAD	-8.7
		ADP-ribose	-6.9
		Apoprotein	-9.5
Z-**32**	TcSir2RP3	NAD	-8.2

		ADP-ribose	-6.9
		apoprotein	-9.4
		NAD	-8.3
E-**32**	TcSir2RP3	ADP-ribose	-7.0
		apoprotein	-9.5

Table S4. Combinatory index (CI) of 12 and BZN

	EC_{50} ratio		Concentration (μM)		CI*
	BZN	**12**	BZN	**12**	
Ratio 1	1/32	1/32	0.8	1.6	6.6
Ratio 2	1/16	1/16	1.5	3.3	13.2
Ratio 3	1/8	1/8	3	6.5	1.02
Ratio 4	1/4	1/4	6	12.5	0.82
Ratio 5	1/2	1/2	12	25	1.14
Ratio 6	1	1	24	50	1.48
Ratio 7	2	2	48	100	2.29

*CI=combinatory index. CI<1 synergism; CI=1 addition/no difference; CI>1 antagonistic

Table S5. Trypanocidal effect of drug combination 17 and BZN

	EC_{50} ratio		Concentration (μM)		CI*
	BZN	**17**	BZN	**17**	
Ratio 1	1/32	1/32	0.8	1.06	0.38
Ratio 2	1/16	1/16	1.5	2.13	0.69
Ratio 3	1/8	1/8	3	4.25	0.73
Ratio 4	1/4	1/4	6	8.5	0.74
Ratio 5	1/2	1/2	12	17	1.25
Ratio 6	1	1	24	34	2.45
Ratio 7	2	2	48	68	3.52

Table S6. Trypanocidal effect of drug combination 32 and BZN

	EC_{50} ratio		Concentration (μM)		CI*
	BZN	**32**	BZN	**32**	
Ratio 1	1/32	1/32	0.8	0.9	0.19

Ratio 2	1/16	1/16	1.5	1.8	0.30
Ratio 3	1/8	1/8	3	3.5	0.82
Ratio 4	1/4	1/4	6	7	0.84
Ratio 5	1/2	1/2	12	14	1.56
Ratio 6	1	1	24	28	1.66
Ratio 7	2	2	48	56	1.82

Table S7. List of complexes used for the docking assessment

PDB ID	Ligand Name	Cofactor Name	Sirtuin
4I5I	4I5	AR6	hSIRT1
5MAT	7KJ	AR6	hSIRT2
5Y0Z	OK9	CNA	hSIRT2
4RMI	3TK	NAD^+	hSIRT2
5D7Q	4I5	NAD^+	hSIRT2
5DY5	5GR	NAD^+	hSIRT2
4JT9	1NS	NAD^+	hSIRT3
4O8Z	BBI	NAD^+	hSIRT3
4RMG	3TE	0.0	hSIRT2
4RMH	3TE	0.0	hSIRT2
5DY4	5GN	0.0	hSIRT2
5MAR	7KE	0.0	hSIRT2
5Y5N	8NO	0.0	hSIRT2
5YQL	A2I	0.0	hSIRT2
5YQM	A2X	0.0	hSIRT2
5YQN	L55	0.0	hSIRT2
5YQO	L5C	0.0	hSIRT2
4BN5	SR7	0.0	hSIRT3
4C7B	BVB	0.0	hSIRT3
4JSR	1NQ	0.0	hSIRT3
4JT8	1NR	0.0	hSIRT3
5H4D	BBI	0.0	hSIRT3
6HOY	TSN	0.0	hSIRT6

Supplementary Figure

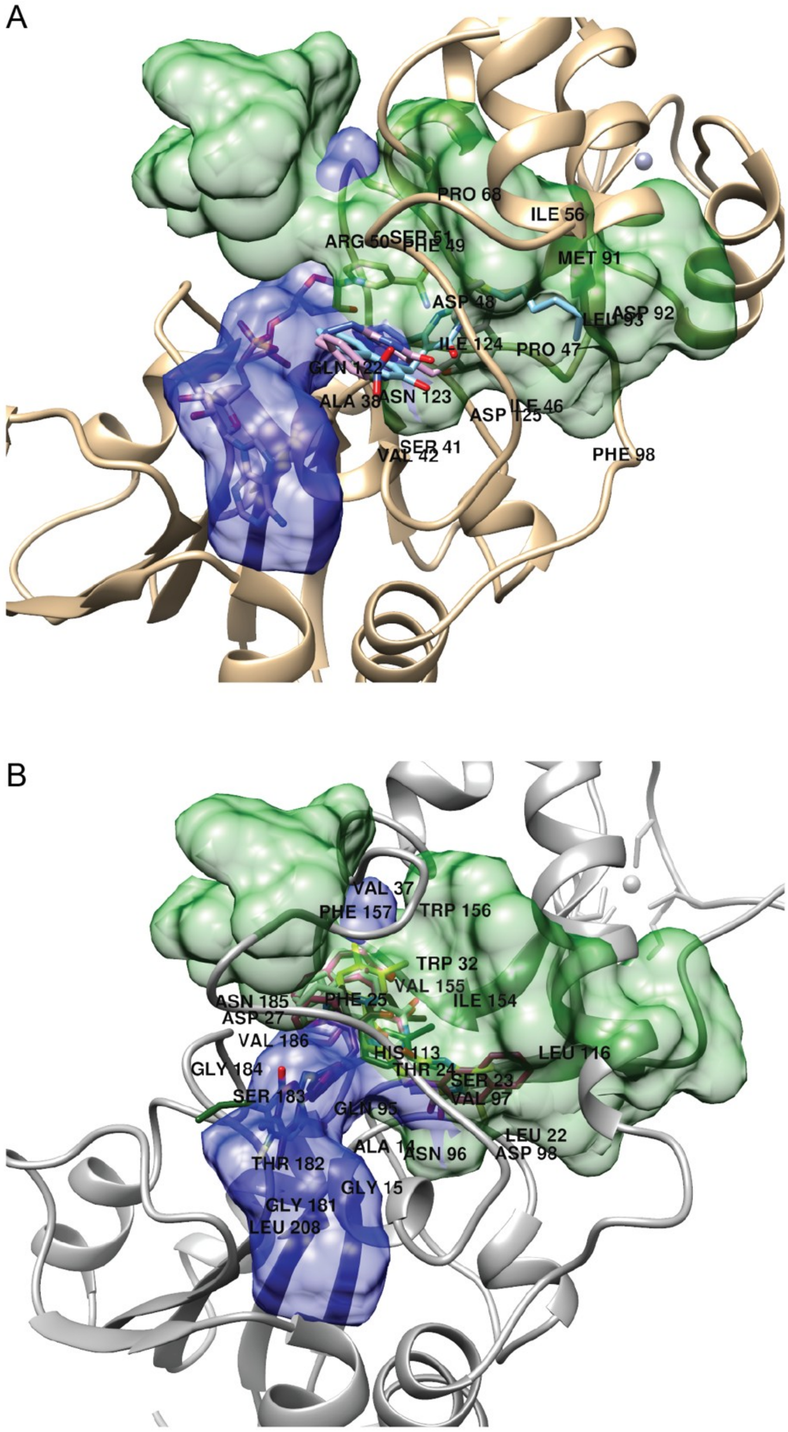

Figure S1. Binding pose of 15 and 17 into TcSir2rp1 modeled structures and poses of 8, 12, 13, 30 and 32 docked into TcSir2rp3, with all the interacting residues.
